# Inter-rater and Intra-rater Reliability in the Radiographic Diagnosis of Growth Arrest in Paediatric Physeal Fractures

**DOI:** 10.1007/s43465-020-00327-9

**Published:** 2021-05-17

**Authors:** Nicole Banting, Emily K. Schaeffer, Jeffrey Bone, Eva Habib, Nikki Hooper, Christopher W. Reilly, Anthony Cooper, Kishore Mulpuri

**Affiliations:** 1 Department of Orthopaedic Surgery, BC Children’s Hospital, 1D.66-4480 Oak Street, Vancouver, BC Canada; 2 Department of Orthopaedics, University of British Columbia, Vancouver, BC Canada

**Keywords:** Reliability, Diagnosis, Radiography, Physeal fractures, Growth arrest

## Abstract

**Background:**

Fractures through the physis account for 18–30% of paediatric fractures and can lead to growth arrest in 5–10% of these cases. Long-term radiographic follow-up is usually necessary to monitor for signs of growth arrest at the affected physis. Given plain radiographs of a physeal fracture obtained throughout patient follow-up, different surgeons may hold different opinions about whether or not early growth arrest has occurred despite using identical radiographs to guide decision-making. This study aims to assess the inter-rater and intra-rater reliability of early growth arrest diagnosis among orthopaedic surgeons given a set of identical plain radiographs.

**Methods:**

A retrospective chart review was conducted on patients aged 2–18 years previously treated for a physeal fracture at a paediatric tertiary care hospital between 2011 and 2018. De-identified anteroposterior (AP) and lateral radiographs of 39 patients from the date of injury and minimum one-year post-injury were administered in a survey to international paediatric orthopaedic surgeons. Each surgeon was asked whether they would diagnose the patient with growth arrest based on the radiographs provided. Surgeons were asked to complete this process again two weeks after the initial review, but using identical shuffled radiographs. Inter-rater and intra-rater reliability was calculated using appropriate kappa statistics.

**Results:**

A total of 11 paediatric orthopaedic surgeons completed the first round of the survey, and 9 of these 11 completed the second round. The inter-rater reliability for the first round was 0.22 [95% CI (0.06, 0.35)] and 0.21 [95% CI (0.02, 0.32)] for the second round. The average kappa for intra-rater reliability was − 0.05 [95% CI (− 0.31, 0.21)]. Comparison by injury side showed no significant variation in diagnosis {*p* = 0.509, OR = 0.90, [95% CI (0.67, 1.22)]}, while comparison by location of injury varied significantly (*p* = 0.003).

**Conclusions:**

Radiographic diagnosis of growth arrest among paediatric orthopaedic surgeons demonstrated ‘fair’ inter-rater agreement and no intra-rater agreement, suggesting critical differences in identifying growth arrest on plain radiographs. Further research is necessary to develop an improved diagnostic approach for growth arrest among orthopaedic surgeons.

**Level of Evidence:**

Diagnostic level III.

## Introduction

The region of the bone known as the physis, or growth plate, contributes to the length and shape of mature long bones. Fractures through the physis account for 18–30% of paediatric fractures and can lead to growth arrest and resulting deformity in 5–10% of these cases [1, 2]. Following a physeal fracture, long-term imaging assessments are often used to monitor for signs of growth arrest at the affected physis. Plain radiographs are readily accessible and provide the initial imaging approach for the evaluation of physeal injuries [3]. Partial or total growth arrest may be identified on plain radiograph by the appearance of bony bridge formation across the physis or through any persistent displacement or angulation for long term [3]. The diagnosis of growth arrest on plain radiographs, combined with further advanced imaging, may be used to guide potential surgical management in patients with a growth disturbance. Therefore, it is clinically important to have a high level of agreement among surgeons when assessing patients with a physeal injury in order to ensure equitable treatment.

Although plain radiographs are widely used by orthopaedic surgeons to assess for initial signs of growth arrest clinically, current studies focus on the assessment of growth arrest using advanced imaging modalities such as magnetic resonance imaging (MRI) and computed tomography (CT), which provide greater ability for detailed identification of bony bridging [4–6]. However, these studies often fail to report inter-rater or intra-rater reliability for the diagnosis of growth arrest using these specific imaging modalities. Additionally, it is difficult to evaluate the clinical relevance of MRI and CT as they are infrequently used in the initial assessment of growth arrest in comparison with plain radiography.

To date, there has been no known study carried out among paediatric orthopaedic surgeons to assess the reliability of growth arrest diagnosis using plain radiographs. The purpose of this study was to examine the inter-rater and intra-rater reliability of growth arrest diagnosis among orthopaedic surgeons given a set of identical plain radiographs.

## Methods

With institutional research ethics board approval, a retrospective radiographic and chart review was conducted on patients, aged 2–18 years, previously treated for a physeal fracture at a paediatric tertiary care hospital between 2011 and 2018. All patients were obtained from a list of participants who were part of a pre-existing, ongoing prospective growth arrest prediction model study at the institution.

A total of 39 patient cases were selected for inclusion. Patients were included if they previously sustained a physeal fracture and had adequate anteroposterior (AP) and lateral radiographs from their date of injury and at a minimum one year post-injury. Patient cases were primarily chosen based on the availability of long-term follow-up (one or two year) radiographs of the injured and contralateral side, which were collected based on the protocol of the institution’s growth arrest prediction model study. Radiographs of the contralateral side were purposely included to provide raters with a greater basis for comparison during diagnosis. Eighteen patient cases had two-year follow-up radiographs in addition to their one-year follow-up radiographs that were used for the study. A variety of growth arrest patients were included in the study, such as patient cases previously diagnosed as having potential growth arrest (11 cases) alongside patient cases without a previously recorded diagnosis of growth arrest (28 cases). Although this may have resulted in a different number of patients diagnosed with growth arrest than may have actually occurred, the agreement regarding the radiographic diagnosis of growth arrest would not have been affected, which was the primary objective of the study.

**Fig. 1:**
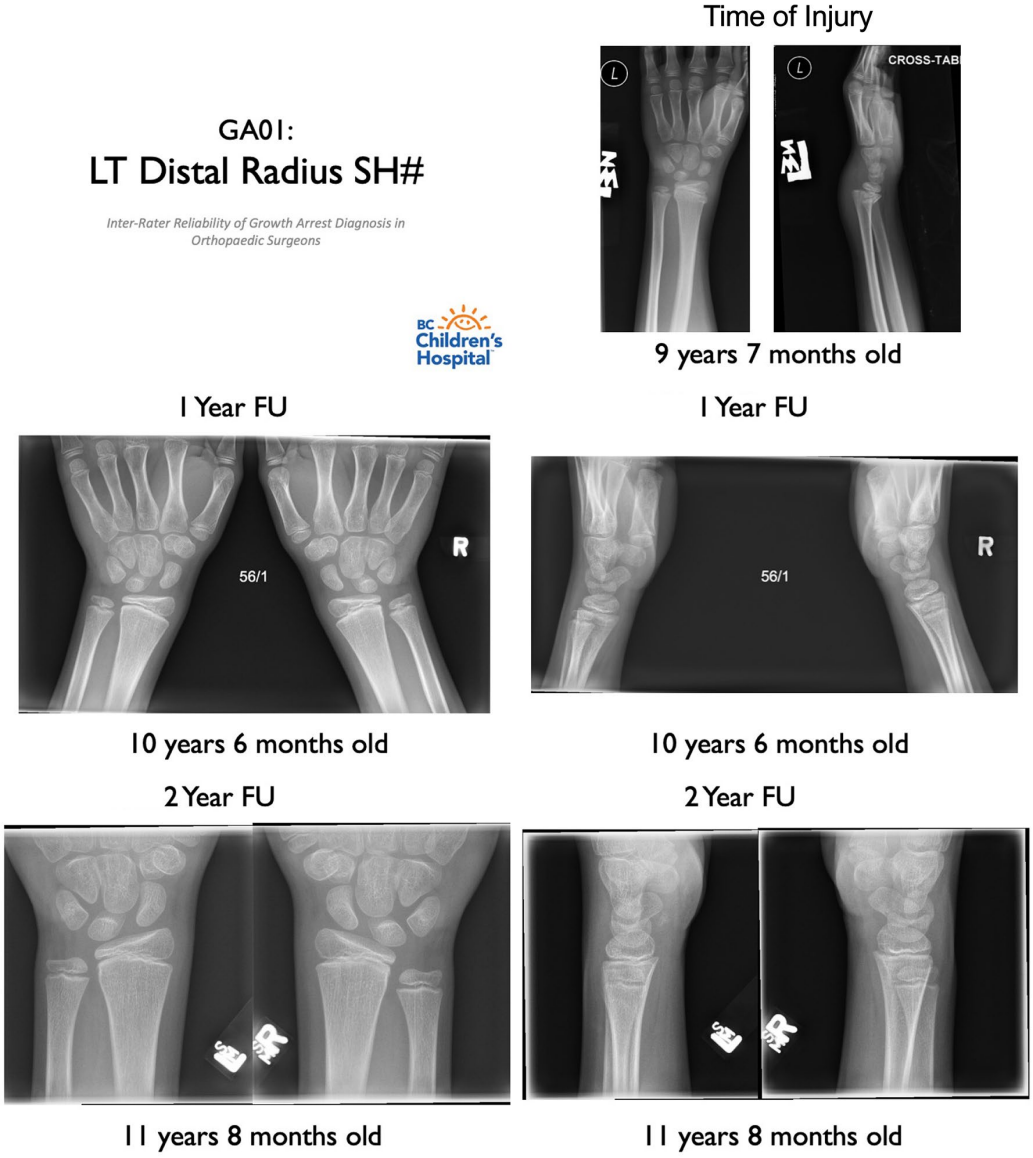
Example of a patient case containing anteroposterior (AP) and lateral radiographs from immediate post-injury and long-term follow-up visits in a patient with a physeal fracture. Each patient case specified the side and anatomical site of the physeal fracture. Immediate post-injury radiographs of the injured side were presented with the date of injury and the patient’s age at injury. Long-term follow-up (one- or two-year) radiographs of the injured and contralateral sides were presented with the date of follow-up and the patient’s age at follow-up. Surgeons were asked whether or not they would diagnose the patient with growth arrest based on the radiographs provided by answering ‘yes’ or ‘no’ on a data collection form

This study was conducted in two parts. First, email invitations to participate in the study were distributed to six fellowship-trained orthopaedic surgeons at a single paediatric tertiary care hospital with extensive experience in managing paediatric physeal fractures. A sample of 39 radiographs of physeal fractures were evaluated by each participating surgeon. De-identified radiograph sets were randomized and distributed to each rater in separate Microsoft PowerPoint presentations (Fig. 1). Each slide in the presentation contained a set of AP and lateral radiographs along with appropriate patient demographic information (patient age, side of injury, length of time since injury). Injured and contralateral limb radiographs from the patient’s long-term follow-up visit(s) were compiled side by side for direct comparison. To investigate the subjective nature of diagnosis, surgeons were intentionally not provided with a defined criteria for diagnosis of growth arrest on the radiographs provided. Each surgeon was asked to rate whether or not they would diagnose the patient with growth arrest by answering ‘yes’ or ‘no’ on a standardized physical data collection form. Additional interpretation of the radiographs was not required as this was beyond the study’s primary objective of assessing diagnostic variability for the presence of growth arrest. To assess for intra-rater reliability, surgeons were provided with identical re-shuffled radiographs after a two-week time interval and were asked to rate whether they would diagnose the patient with growth arrest a second time.

Second, invitations to participate in the study were extended to six paediatric orthopaedic surgeons from international centres who primarily treat paediatric trauma patients. The same set of radiographs were administered in the form of a survey through Research Electronic Data Capture (REDCap) software [7] and followed the format of the PowerPoint presentations. The surveys were administered in two rounds following the same procedures described above. For both rounds, surgeons were asked to select whether or not they would diagnose the patient with growth arrest by answering ‘yes’ or ‘no’ on the REDCap survey.

Inter-rater and intra-rater reliability of growth arrest diagnosis was calculated using appropriate kappa statistics, and 95% confidence intervals (CI) were computed based on 1000 bootstrap resamples. Specifically, Fleiss’ kappa was calculated for each of the two rounds in order to assess the inter-rater reliability among surgeons [8]. Cohen’s kappa statistic was determined for each individual surgeon comparing scores between the first and second rounds for intra-rater reliability [9]. Interpretation of the kappa values was based on the criteria according to Cohen [10]: values 0.81–1.00 indicate ‘almost perfect’ agreement, 0.61–0.80 ‘substantial’, 0.41–0.60 ‘moderate’, 0.21–0.40 ‘fair’, 0.01–0.20 ‘none to slight’, and values less than or equal to zero as ‘no agreement’. Growth arrest diagnosis was compared both by injury side and by injury type via mixed-effects logistic regression with a random intercept for ‘surgeon’ [11]. Statistical significance of injury side and type of injury was based on the likelihood ratio test. All analyses were carried out using R 3.5.3 [12].

## Results

### Demographics

A total of six paediatric orthopaedic surgeons from the primary institution and six paediatric orthopaedic surgeons from various international centres, known by the senior author (KM), were invited to participate in the study in February 2019. All participating raters were fellowship-trained, board-certified paediatric orthopaedic surgeons in active practice. Of those invited, one surgeon partially completed the first survey round and 11 surgeons (7 from Canada, 2 USA, 1 Australia, 1 New Zealand, and 1 India) fully completed the first survey round. All responses from the first round of surveys were registered in late February 2019. Nine of the 11 original respondents completed the second round, with all responses registered in March 2019.

A sample of 39 radiographs of physeal fractures were evaluated by each participating surgeon. The types of physeal fractures included: distal humerus (6), distal radius (14), proximal radius (1), distal femur (1), distal tibia (15), and distal fibula (2). There were 20 left-sided injuries and 19 right-sided injuries.

### Total Growth Arrest Diagnoses

Comparison by injury side showed no significant variation in diagnosis {*p* = 0.5087, [OR = 0.90, 95% CI (0.67, 1.22)]}. In both rounds of the study, left-sided injuries were diagnosed with growth arrest a total of 152 times (36%) in comparison with a diagnosis of no growth arrest 267 times (64%). Similarly, right-sided injuries were diagnosed a total of 132 times (34%) with growth arrest and 258 times (66%) without growth arrest.

Comparison by injury type, however, showed significant variation in growth arrest diagnosis (*p* = 0.003) and predicted the surgeons’ responses (Table 1). Injuries of the right distal humerus were the most common injury type diagnosed with growth arrest (49%), while injuries of the right proximal radius were the least commonly diagnosed injury type (14%).

**Table 1:** Comparison of diagnosis responses based on injury type (reference is to left distal femur)

Description of injury	Number of patient cases	Number diagnosed with no growth arrest	Number diagnosed with growth arrest	Odds ratio	95% CI	*p* value
Left distal femur	1	14 (67%)	7 (33%)	Reference	Reference	Reference
Left distal humerus	3	41 (65%)	22 (35%)	1.07	(0.36, 3.14)	0.91
Left distal radius	8	118 (63%)	70 (37%)	1.2	(0.45, 3.2)	0.72
Left distal tibia	7	94 (64%)	53 (36%)	1.13	(0.42, 3.06)	0.81
Right distal fibula	2	32 (78%)	9 (22%)	0.55	(0.16, 1.82)	0.32
Right distal humerus	3	31 (51%)	30 (49%)	2.06	(0.71, 6.02)	0.19
Right distal radius	4	73 (71%)	30 (29%)	0.82	(0.29, 2.29)	0.7
Right distal tibia	8	104 (63%)	60 (37%)	1.17	(0.44, 3.17)	0.75
Right proximal radius	1	18 (86%)	3 (14%)	0.32	(0.07, 1.49)	0.15

The number of cases diagnosed with or without growth arrest is based on the responses of 12 surgeons over both rounds of the survey. One surgeon partially completed the first survey round, and 11 surgeons fully completed the first survey round, while nine of the 11 original respondents fully completed the second survey round

### Inter-rater Reliability

The inter-rater reliability for growth arrest diagnosis showed significant variability among surgeons (Table 2). For the first round, the Fleiss’ kappa across 11 responding surgeons was 0.22 [95% CI (0.06, 0.35)], indicating fair agreement. The second round indicated fair agreement among the nine responding surgeons with a similar Fleiss’ kappa of 0.21 [95% CI (0.02, 0.32)].

**Table 2:** Inter-rater Fleiss’ kappa statistics and interpretation

Round	Fleiss’ kappa	95% CI	*p* value	Agreement
1	0.22	(0.06, 0.35)	< 0.001	Fair
2	0.21	(0.02, 0.32)	< 0.001	Fair

### Intra-rater Reliability

Intra-rater reliability of growth arrest diagnosis between rounds 1 and 2, which contained the same 39 patient cases arranged in a different order, showed significant variability (Table 3). In the second round, some surgeons had missing responses and were therefore excluded. The average weighted kappa for the first and second rounds was − 0.05 [95% CI − 0.31, 0.21], indicating no agreement.

**Table 3:** Intra-rater kappa statistics and overall average

Rater	Kappa	95% CI	Agreement
1	0.04	(− 0.27, 0.35)	Poor
2	− 0.04	(− 0.35, 0.27)	Poor
3	− 0.22	(− 0.52, 0.07)	Poor
4	− 0.18	(− 0.47, 0.12)	Poor
5	NA	(NA, NA)	NA
6	NA	(NA, NA)	NA
7	0.18	(− 0.14, 0.49)	Poor
8	− 0.16	(− 0.41, 0.10)	Poor
9	− 0.1	(− 0.39, 0.19)	Poor
10	0.07	(− 0.25, 0.38)	Poor
11	N/A	(NA, NA)	NA
12	− 0.07	(− 0.15, 0.00)	Poor
Average	− 0.05	(− 0.31, 0.21)	Poor

## Discussion

The results of this study demonstrate ‘fair’ inter-rater agreement and no intra-rater agreement with regard to growth arrest diagnosis among paediatric orthopaedic surgeons. These findings suggest critical differences in diagnosis, illustrating the difficulty in identifying growth arrest on plain radiographs. This lack of consistency is clinically relevant, given that a diagnosis of growth arrest is used to guide treatment decisions and to prevent deformity and limb length discrepancy in children with physeal fractures. Failure to have consistent diagnoses between patients may result in differing outcomes between patients presenting with similar conditions.

A limited number of studies have measured the reliability of radiographic assessment for physeal injuries. Tzavellas et al. recently conducted a reliability study of Salter–Harris classification using radiographs of physeal injures and found moderate agreement between raters with regard to injury classification [13]. However, it was beyond the purpose of Tzavellas’ study to look at the diagnosis of growth arrest using long-term radiographs, which was the objective of our study. Studies that have assessed the interpretation of growth arrest often discuss MRI and CT imaging, which provide greater detail on the size, location, and shape of the bone bridge compared to conventional radiography, which has yet to be addressed in the current literature [4–6]. Due to the routine use and accessibility of plain radiography, our study focused on the radiographic diagnosis of growth arrest; however, future work should also consider the reliability of growth arrest diagnosis on advanced imaging. At present, there are no formal studies that report inter-rater or intra-rater reliability for the diagnosis of growth arrest using any of these imaging modalities.

There are several limitations to this study. First, no standard definition of growth arrest was given to participating surgeons before they were asked to identify the presence of growth arrest from the radiographs provided. A concrete diagnostic definition for growth arrest, typically specified by the appearance of a bony bridge across the physis, was intentionally not provided in order to investigate the subjective nature of diagnosis for physeal injuries; this could have led to some surgeons reporting a diagnosis of growth arrest due to factors other than the presence of a bone bridge on the radiograph, such as whether they believed the outcome of the injury would be clinically significant. This may indicate the need for a standardized definition and method of diagnosis for future studies involving the radiographic diagnosis of growth arrest. The inclusion of physeal fractures regardless of the site of injury could also be a weakness as the selected cases may not be an accurate representation of possible fracture types. Due to the fact that the selection of patient cases was based on the availability of contralateral-side radiographs from a minimum of a one-year follow-up for comparison, it was difficult to include a uniform distribution of fractures based on the anatomic site of injury. This study included only one patient case with a physeal fracture at the proximal end of the long bone; the rest of the patient cases represented fractures at the distal end of the long bones. Distal radius and distal tibia fractures also outnumbered other fracture types, which may have influenced the study results for the number of growth arrest diagnoses. This study would have been strengthened by greater inclusion of injuries from other anatomic sites, including those that have historically resulted in frequent growth disturbances such as the distal femur [14]. One possible way to achieve this would be to include patient cases containing radiographs from the date of injury and long-term follow-up, but without the contralateral-side image, in order to obtain a greater distribution of fracture types. Further work is required using an equal number of injury types to examine differences in diagnosis within stratified subgroups. Finally, this study could have benefitted from a larger sample of participating surgeons. There is some bias with regard to selection of the raters, all of whom were chosen by the senior author. It is possible that other surgeons who did not participate in this study may demonstrate a different level of inter-rater and intra-rater reliability than was found in this study.

This study lays the groundwork for a variety of future studies. Building on Tzavellas’ work [13], testing the inter-rater and intra-rater reliability of the Salter–Harris classification (the most widely followed classification for physeal fractures) on a large number of radiographs and raters would be useful to explore potential variabilities in fracture classification. While this study asked surgeons to diagnose the presence of growth arrest on radiographs, it would also be beneficial to assess the subjective criteria that each surgeon used to determine the diagnosis of growth arrest. Modification of future reliability studies could include the collection of data pertaining to individual assessment of bony bridging or subjective indications for diagnosis. In addition, future studies could examine the diagnostic accuracy of plain radiography and advanced imaging modalities like MRI and CT (i.e., which radiographically diagnosed growth arrests result in real growth arrests requiring treatment). It is also well known that growth disturbances can occur after good anatomic reduction in Salter–Harris Type III, IV, and V physeal fractures where the growth plate has likely been damaged at the moment of impact [15]. A retrospective study to evaluate the timing of reduction and its role in the development of growth arrest after injury may be of worthwhile investigation.

The current study illustrates variability among surgeons when diagnosing growth arrest on plain radiographs. These discrepancies, though interesting, are not necessarily expected to change clinical practice since further advanced imaging is compulsory when there are queries related to diagnosis of physeal injuries. However, these findings may serve as an initial step towards highlighting disagreement among surgeons with regard to radiographic diagnosis of growth arrest. A more robust study that includes a greater number of patient cases and comparisons to advanced imaging may further investigate this disagreement in diagnosis. At the very least, this study demonstrates the need for a standardized definition of growth arrest and a greater need for improved methods of identifying growth arrest on plain radiographs.

## Conclusion

This study provides evidence for a low level of both inter-rater and intra-rater reliabilities among paediatric orthopaedic surgeons in the radiographic diagnosis of growth arrest following a physeal injury. These findings suggest differences with regard to the interpretation of growth arrest on plain radiographs at the patient’s one- or two-year follow-up visit. Potential future studies can look at the reliability of growth arrest diagnosis in plain radiographs with regard to Salter–Harris classification, levels of clinical experience between raters, as well as reliability of advanced imaging methods, such as MRI and CT. Further understanding of the levels of agreement in growth arrest diagnosis may help identify potentially critical variation during diagnosis, and as such, encourage development of an improved diagnostic approach in order to optimize clinical and functional outcomes in patients with physeal fractures.

## References

[1] Mizuta T, Benson WM, Foster BK, Morris LL (1987). Statistical analysis of the incidence of physeal injuries. Journal of Pediatric Orthopedics.

[2] Mann DC, Rajmaira S (1990). Distribution of physeal and nonphyseal fractures in 2650 long-bone fractures in children aged 0–16 years. Journal of Pediatric Orthopedics.

[3] Wang DC, Deeney V, Roach JW, Shah AJ (2015). Imaging of physeal bars in children. Pediatric Radiology.

[4] Ecklund K, Jaramillo D (2002). Patterns of premature physeal arrest: MR imaging of 111 children. AJR American Journal of Roentgenology.

[5] Wioland M, Bonnerot V (1993). Diagnosis of partial and total physeal arrest by bone single-photon emission computed tomography. Journal of Nuclear Medicine.

[6] Sailhan F, Chotel F, Guibal AL, Gollogly S, Adam P, Bérard J, Guibaud L (2004). Three-dimensional MR imaging in the assessment of physeal growth arrest. European Radiology.

[7] Harris PA, Thielke R, Taylor R, Payne J, Gonzalez N, Conde JG (2009). Research Electronic Data Capture (REDCap)—a metadata-driven methodology and workflow process for providing translational research informatics support. Journal of Biomedical Informatics.

[8] Fleiss JL (1971). Measuring nominal scale agreement among many raters. Psychological Bulletin.

[9] Cohen J (1968). Weighted kappa: nominal scale agreement provision for scaled disagreement or partial credit. Psychological Bulletin.

[10] McHugh ML (2012). Interrater reliability: the kappa statistic. Biochem Med (Zagreb).

[11] Bates D, Maechler M, Bolker B, Walker S (2015). Fitting Linear Mixed-Effects Models Using lme4. Journal of Statistical Software..

[12] R Core Team (2018) R: a language and environment for statistical computing. R Foundation for Statistical Computing, Vienna. https://www.R-project.org

[13] Tzavellas AN, Kenanidis E, Potoupnis M, Pellios S, Tsiridis E, Sayegh F (2016). Interobserver and intraobserver reliability of Salter-Harris classification of physeal injuries. Hippokratia..

[14] Ecklund K, Jaramillo D (2001). Imaging of growth disturbance in children. Radiologic Clinics of North America.

[15] Dabash S, Prabhakar G, Potter E, Thabet AM, Abdelgawad A, Heinrich S (2018). Management of growth arrest: current practice and future directions. Journal of Clinical Orthopedics and Trauma.

